# Decoding the mechanism of Eleutheroside E in treating osteoporosis via network pharmacological analysis and molecular docking of osteoclast-related genes and gut microbiota

**DOI:** 10.3389/fendo.2023.1257298

**Published:** 2023-11-07

**Authors:** Tianyu Zhou, Yilin Zhou, Dongdong Ge, Youhong Xie, Jiangyan Wang, Lin Tang, Qunwei Dong, Ping Sun

**Affiliations:** ^1^ Department of Endocrinology, The First Affiliated Hospital of Guangdong Pharmaceutical University, Guangzhou, China; ^2^ Department of Orthopedics, The First Affiliated Hospital of Guangdong Pharmaceutical University, Guangzhou, China; ^3^ Department of Orthopedics, Yunfu Hospital of Traditional Chinese Medicine, Yunfu, China

**Keywords:** Eleutheroside E (EE), postmenopausal osteoporosis (PMO), network pharmacology, molecular docking, gut microbiota

## Abstract

**Objective:**

Eleutheroside E (EE) is an anti-inflammatory natural compound derived from the edible medicinal herb *Acanthopanax senticosus*. This study aims to investigate the underlying mechanism of the anti-osteoporosis action of EE through network pharmacology, molecular docking and gut microbiota.

**Materials and methods:**

Network pharmacology was used to explore the potential core targets and main pathways mediated by EE in osteoporosis (OP) treatment. Molecular docking was exploited to investigate the interactions between the active anti-OP compounds in EE and the potential downstream targets. Following the multi-approach bioinformatics analysis, ovariectomy (OVX) model was also established to investigate the *in vivo* anti-OP effects of EE.

**Results:**

The top 10 core targets in PPI network were TP53, AKT1, JUN, CTNNB1, STAT3, HIF1A, EP300, CREB1, IL1B and ESR1. Molecular docking results that the binding energy of target proteins and the active compounds was approximately between −5.0 and −7.0 kcal/mol, which EE has the lowest docking binding energy with HIF1A. Enrichment analysis of GO and KEGG pathways of target proteins indicated that EE treatment could potentially alter numerous biological processes and cellular pathways. *In vivo* experiments demonstrated the protective effect of EE treatment against accelerated bone loss, where reduced serum levels of TRAP, CTX, TNF-α, LPS, and IL-6 and increased bone volume and serum levels of P1NP were observed in EE-treated mice. In addition, changes in gut microbiota were spotted by 16S rRNA gene sequencing, showing that EE treatment increased the relative abundance of *Lactobacillus* and decreased the relative abundance of *Clostridiaceae*.

**Conclusion:**

In summary, these findings suggested that the characteristics of multi-target and multi-pathway of EE against OP. *In vivo*, EE prevents the onset of OP by regulating gut microbiota and inflammatory response and is therefore a potential OP drug.

## 1 Introduction

Osteoporosis (OP) is a common systemic bone disease that can lead to increase susceptibility to fractures due to a variety of reasons, such as decreased bone mineral density, deteriorated bone microstructure, increased bone fragility, and so on (1). Postmenopausal osteoporosis (PMO) is one of the most common forms of OP, which is a metabolic disease caused by the decline of ovarian function and estrogen levels in postmenopausal women (2). At present, most of the treatments for OP are chemical drugs that regulate bone metabolism, which are often associated with adverse effects, such as renal injury and joint pain (3, 4). Therefore, proposing novel therapeutics with high clinical efficacy and milder side effects is an important field of OP research.

Traditional Chinese medicine (TCM) is clinically more compelling over current anti-OP medications due to its safety and effectiveness. And TCM has a long history acting as a complementary and alternative treatment for OP patients (5, 6). *Acanthopanax senticosus* (*A. senticosus*) which has been extensively used as nutritious food and oriental medicine in Asia belongs to the *Araliaceae* family and is widely distributed in China, Korea, and Japan. *A. senticosus* has been used to treat rheumatoid arthritis, diabetes, and hypertension for a long time and exhibited *in vivo* immunomodulating activities (7–9). Eleutheroside E (EE) is an essential active constituent derived from *A. senticosus*. EE was found ameliorate arthritis severity by suppressing inflammatory cytokine release in arthritis mice model (10). However, the underlying mechanism of the anti-OP action of EE has not been fully elucidated.

In the past decade, ever-growing evidence has shown a strong association between the gut microbiota and many human diseases (11, 12). For instance, the gut microbiota can regulate the function of the human intestinal endocrine system, intestinal nervous system, intestinal permeability (13). Interestingly, recent findings have linked the gut microbiota to the development and progression of OP (14, 15). Therefore, identifying novel anti-OP drugs that act on gut microbiota may offer a new solution to overcome those unwanted effects observed in conventional OP treatments. Network pharmacology has been extensively used to elucidate the mechanism of action of TCM. In this study, we investigated the effect of EE on osteoclast-related genes through network pharmacology, molecular docking and verified its protective effect against excessive bone loss on OVX mice, providing new insights into the mechanism of action of TCM.

## 2 Materials and methods

### 2.1 Target acquisition of EE

The active components of EE were extracted from the TCMSP (https://tcmspw.com/tcmsp.php). The compounds were screened according to oral bioavailability (OB) ≥30% and drug likeness (DL) ≥0.18 (16). SDF format of the 2D structures of the compounds was obtained by entering the CAS numbers of EE in PubChem (https://pubchem.ncbi.nlm.nih.gov). Then it was imported into STP (http://swisstargetprediction.ch/) to obtain the predicted targets of the chemical components. The active ingredient targets were obtained after screening by probability>0. Target information from the STP and TCMSP database was integrated to get the potential targets of EE components (17). UniProt (https://www.uniprot.org) was used to convert each target into genes (18). EE effects on osteoclast for potential target procurement. OC-related targets were obtained from the GeneCards (https://www.genecards.org), OMIM (https://www.omim.org), and TTD databases (http://db.idrblab.net/ttd/) with osteoclast differentiation and osteoclastogenesis as the keywords. GeneCards were filtered above the median relevance score (19). The intersection of EE components and OC-related targets was plotted using an online Venn analysis tool.

### 2.2 PPI network analysis and critical target acquisition

Protein-protein interaction (PPI) underlies most biological processes in living cells and is essential for understanding cellular physiology in normal and disease states (20). The potential target genes of EE action on osteoclast were uploaded to the STRING database (https://cn.string-db.org/). The protein species was set to “Homo Sapiens” and the interaction score was set to 0.4. Meanwhile the unconnected nodes in the network were hidden to obtain and the interaction results were saved as TSV format files. The files were imported into Cytoscape software to draw PPI network diagrams, and topological parameters were analyzed by applying the network analyzer of Cytoscape’s built-in network analysis plug-in (21). According to the degree of target genes, the greater the degree was, the more critical role it played in the process of EE acting on osteoclast.

### 2.3 Enrichment analysis of GO and KEGG pathways

To further elaborate the mechanism of EE action on osteoclast, Metascape (https://metascape.org) was used to perform GO term enrichment analysis and KEGG pathway enrichment analysis on the intersection targets (22). Microsign online graphing software was used to visualize GO functional enrichment and KEGG pathway analysis data. The species option was set to “Homo Sapiens”, and the analysis about the cell components, molecular functions, biological process, and signaling pathways involving the targets was conducted. Pathways and biological processes for which *P* < 0.05 were extracted.

### 2.4 Molecular docking

The obtained core targets were molecular docking to the core components. The protein structures of the core targets were downloaded from the PDB database (https://www.rcsb.org/), and then the core proteins were dehydrated and delighted with PyMol 2.4.0 software. The structures of the core components were retrieved by PubChem, downloaded in SDF format, and converted to pdb format with OpenBabel 3.1.1. AutoDock 4.2.6 software was used to routinely process protein receptors and small molecule ligands and save them in pdbqt format. Molecular docking and binding energy calculation scripts were run using AutoDock Vina 1.1.2 (23), and some results were visualized using PyMol software (24).

### 2.5 Animals and materials

EE was purchased from CHENGDU MUST BIO-TECHNOLOGY CO.,LTD. Twenty C57BL/6J female mice (7–week-old; 18 ± 1g) were acquired from the Guangdong Medical Laboratory Animal Center. These animals were placed in a 12 hours light/dark cycle. After acclimation for 7 days, all mice were randomly divided into four groups (n=5 per group): Sham group, OVX group, EE-L (20 mg/kg) group, and EE-H (40 mg/kg) group. All animals had free access to food and water. EE was dissolved in 1% carboxymethyl cellulose sodium (CMC-Na). Conversely, the Sham and OVX group received intragastric administration of equal volume CMC-Na. After 12 weeks, mice were sacrificed and collected their left femur, serum and feces. The Ethics Committee of Guangdong Pharmaceutical University had reviewed and approved the experimental protocol according to the Guide for the Care and Use of Laboratory Animals.

### 2.6 Micro-CT analysis and ELISA tests

Micro-CT (SCANCO uCT-100 detector, Switzerland) was used to scan the morphological structure of the left distal femur of mice, and the bone trabeculae in the area 0.8 mm above 0.5 mm from the growth plate were selected as the region of interest (ROI) for 3D reconstruction. Bone mineral density (BMD), bone volume fraction (BV/TV), bone surface/bone volume (BS/BV), number of trabeculae (Tb.N) and trabecular separation (Tb.Sp) of the ROI were analyzed with evaluation analysis software. The analysis ranges were kept consistent for all specimens.

The serum was obtained from anesthetized animals by retroorbital puncture at the end of the study. The levels of TRAP, CTX, P1NP, TNF-α, LPS and IL-6 were measured by ELISA according to the manufacturer’s instructions.

### 2.7 Gut microbiota analysis

After collecting the mice feces, the feces were placed in sterile cryopreservation tubes, rapidly frozen in liquid nitrogen, and stored in an ultra-low temperature refrigerator at -80°C for gut microbiota diversity analysis. The E.Z.N.A.®soil DNA kit (Omega Bio-Tek, Norcross, GA, U.S.) was used to extract DNA from each mouse fecal sample, and the extracted DNA was detected by 1% agarose gel electrophoresis. Then all primers were designed based on the conserved region. 338F (5′-ACTCCTACGGGAGGCAGCA-3′) and 806R (5′-GGACTACHVGGGTWTCTAAT-3′) were used for PCR amplification of the V3-V4 region of the 16S rRNA gene to perform the illumina deep sequencing. PCR products were detected by 2% agarose gel electrophoresis. The purified PCR products were sequenced using the NEXTFLEX®Rapid DNA-Seq kit. The PCR products were sequenced through Illumina’s Miseq PE300 platform (Shanghai Majorbio Biopharm Technology, China).

### 2.8 Statistical analysis

All data were represented as mean *± SD*. The *ANOVA* test was used for the data with normal distribution, and the *Mann–Whitney* test was used to analyze data that did not meet the assumptions of the *ANOVA* (*SPSS*, version 26.0). When *P*<0.05, the differences were considered to be statistically significant.

## 3 Results

### 3.1 Prediction of component targets and gene targets

**Figure 1:**
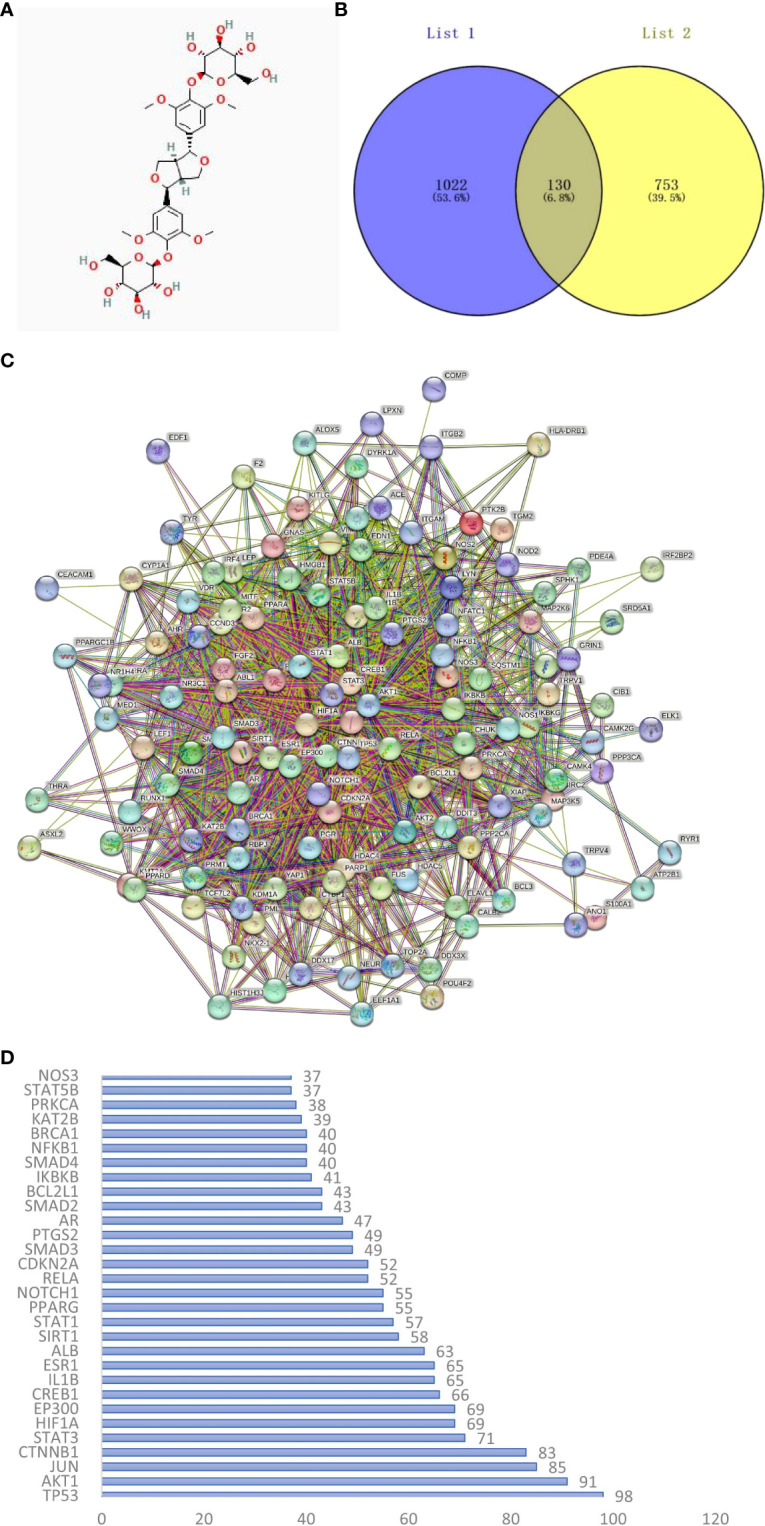
**(A)** The chemical structure of EE was provided by the PubChem database (CID:71312557). **(B)** Venn diagram of osteoclast-related targets (List 1) and EE-related targets (List 2). **(C)** PPI network of overlapping genes between EE and osteoclast was obtained from the STRING database. **(D)** Ranking the top 30 targets in the PPI network in degree.

The 2D structure of EE is shown in **Figure 1A**. We retrieved 15 herbal component targets through the TCMSP and STP databases. All the targets were transformed into 883 genes using the UniProt database. 1152 target genes related to osteoclast differentiation and osteoclastogenesis were captured from the GeneCards, OMIM, and TTD databases. 130 potential targets of EE action on osteoclast were selected by taking the overlap through the Venn online platform (**Figure 1B**).

### 3.2 Construction of PPI networks and acquisition of core proteins

The interaction relationships of potential EE targets affecting osteoclast were obtained from the STRING database (**Figure 1C**), in which the PPI network displayed 130 functional proteins with 1686 interactions. With the node degree as the evaluation parameter, where a higher node degree indicates a more important role in the PPI network. The top 10 core targets in terms of the node degree are TP53, AKT1, JUN, CTNNB1, STAT3, HIF1A, EP300, CREB1, IL1B, and ESR1 (**Figure 1D**).

### 3.3 GO and KEGG pathway enrichment analysis

**Figure 2:**
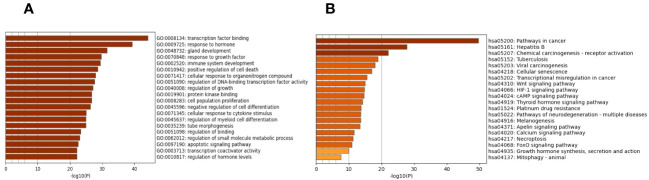
GO functional annotation and KEGG pathway enrichments. **(A)** The plots of the 20 most significant biological process based on GO enrichment analysis. **(B)** The top 20 enriched KEGG terms of signaling pathways. The X-axis and Y-axis stand for the gene ratios and full names of the processes, respectively.

The biological process, cellular composition, and molecular function were selected by GO analysis of 130 common targets. The GO enrichment analysis showed that those selective targets are mainly involved in processes of transcription factor binding (GO:0008134), response to hormone (GO:0009725), gland development (GO:0048732), response to growth factor (GO:0070848) (**Figure 2A**). To further explore the potential pathways of EE in the treatment of OP, KEGG analysis showed that common targets are mainly enriched in the Wnt signaling pathway (has:04310), HIF-1 signaling pathway (has:04066), cAMP signaling pathway (has:04024) and Calcium signaling pathway (has:04020) (**Figure 2B**).

### 3.4 Molecular docking

**Figure 3:**
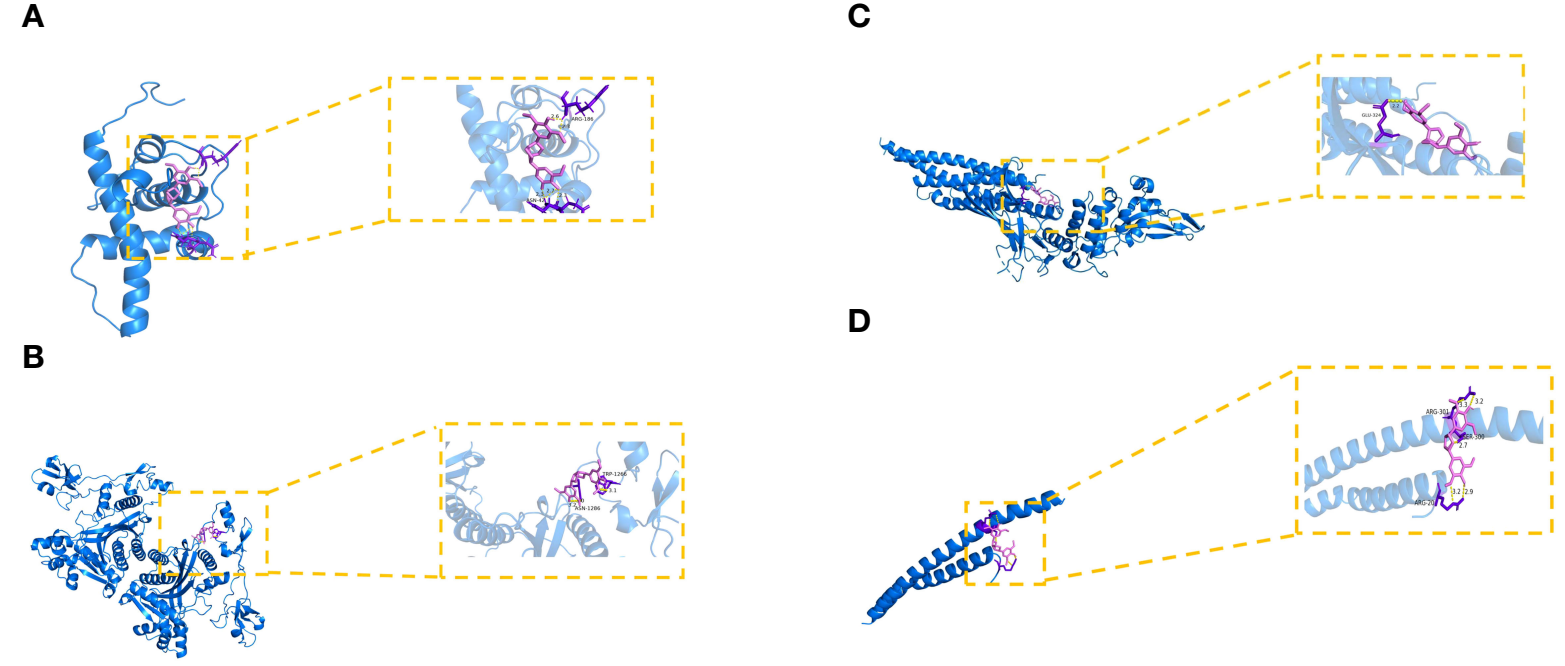
Molecular docking of hub targets and active components of EE. **(A) **The binding modes of EE to HIF1A. **(B)** The interaction modes of EE with EP300. **(C)** The binding modes of EE to STAT3. **(D)** The interaction modes of EE with CREB1.

To further look into the molecular basis of this compound and identify associated mechanistic targets, EE was selected to perform molecular docking with the top 10 core targets predicted by the PPI network analysis. The absolute values of the docking score indicate the affinity of the components with the targets and the stability of the component-target conformation. An absolute value greater than 5.0 indicates a good binding, and greater than 7.0 indicates a strong binding (**Table 1**) (25, 26). The binding energy of target proteins and the active compounds is approximately between −5.0 and −7.0 kcal/mol, which means that EE bind well to 10 core target proteins. Comprehensive analysis shows that the docking scores of EE with HIF1A have the highest absolute value among the targets. We chose the top 4 target proteins macromolecules and small compound molecules with the best docking affinity for visualization by PyMol, which were HIF1A, EP300, STAT3 and CREB1 (**Figure 3**).

**Table 1:** Docking results of core target proteins and core active components.

Targets	PDB ID	Affinity(kcal/mol)
TP53	6MY0	-5.4
AKT1	2UZR	-5.7
JUN	1JUN	-5.2
CTNNB1	2Z6H	-6.1
STAT3	6NJS	-6.4
HIF1A	1L3E	-6.9
EP300	8GZC	-6.6
CREB1	5ZK1	-6.4
IL1B	1HIB	-5.9
ESR1	4N1Y	-6.2

### 3.5 EE alleviated bone loss in OVX mice

**Figure 4:**
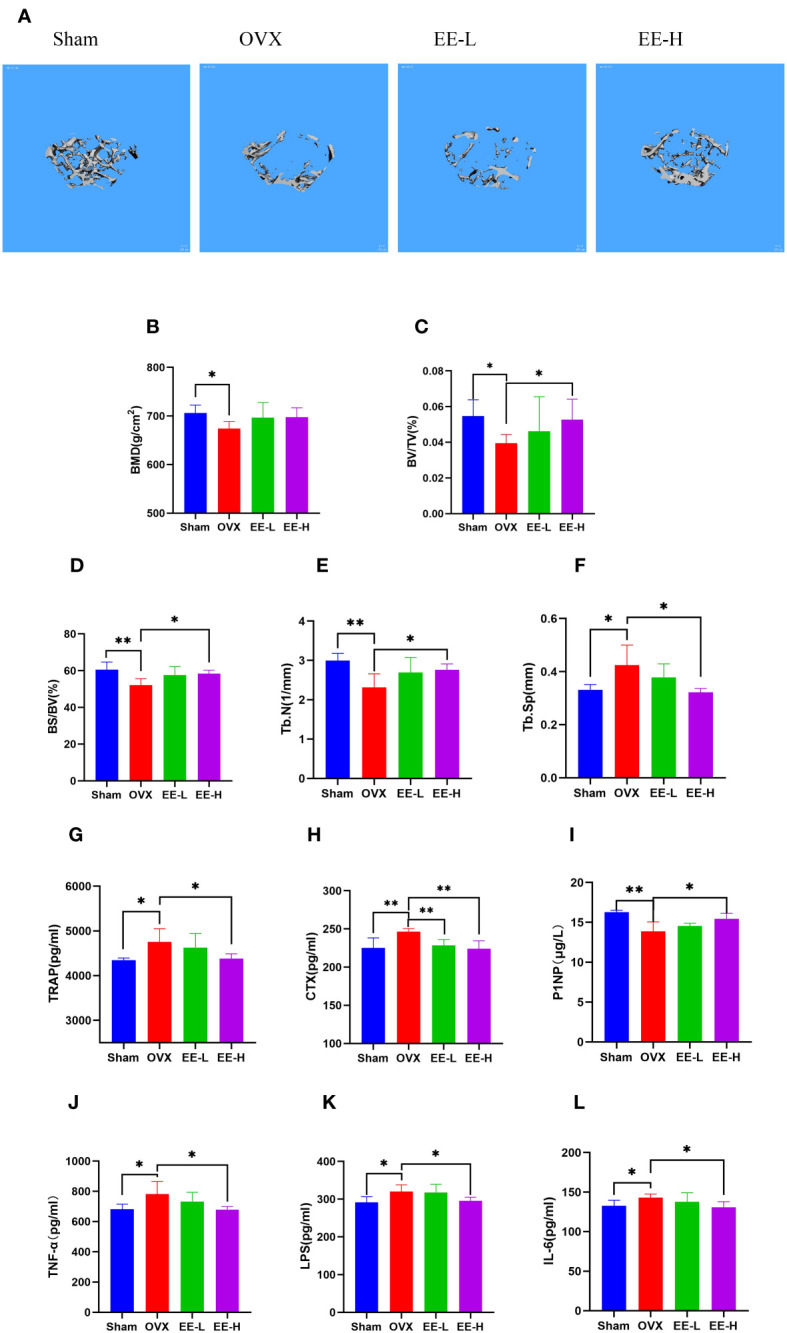
EE treatment improved OVX-induced bone loss *in vivo*. **(A)** Representative μCT images indicated that the bone loss was prevented by EE (scale bar=100 μm). **(B–F)** μCT quantitative parameters for bone microstructure including BMD, BV/TV, BS/BV, Tb.N and Tb.Sp. **(G–L)** Serum levels of TRAP, CTX, P1NP, TNF-α, LPS, and IL-6 in different groups were detected by ELISA. (n=5). *p<0.05, **p<0.01.

The reconstructed 3D images confirmed that OVX underwent significant bone loss, which was attenuated by EE treatment (**Figure 4A**). The values of BMD, BV/TV, BS/BV, and Tb.N were decreased significantly in the OVX compared with the Sham, although the value of Tb.Sp was increased significantly (*P*<0.05, *P*<0.01). The levels of BV/TV, BS/BV, and Tb.N were increased significantly, while Tb.Sp was presented an opposite trend in EE-H (*P*<0.05). The value of BMD was enhanced but not significantly different (*P*>0.05) (**Figures 4B–F**).

Serum P1NP levels of mice in the OVX was significantly decreased compared with the Sham (*P*<0.01), a sharp increased in P1NP by EE (*P*<0.05), moreover, the contents of TRAP, CTX, TNF-α, LPS and IL-6 were significantly increased in the OVX (*P*<0.05, *P*<0.01). Treatment of EE reversed the increased serum levels of TRAP, CTX, TNF-α, LPS and IL-6 (*P*<0.05, *P*<0.01) (**Figures 4G–L**).

### 3.6 EE regulation of gut microbiota in OVX mice

**Figure 5:**
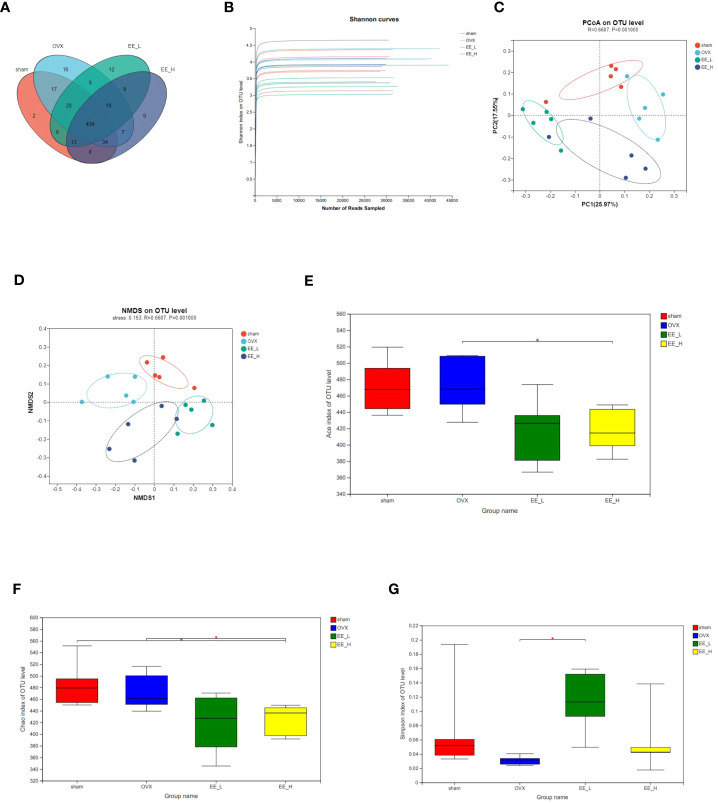
EE changed the gut microbiota community composition in OVX mice. **(A)** The OTU data of each group was represented by Venn diagram. **(B)** Shannon curves. **(C)** PCoA on OTU level. **(D)** NMDS on OTU level. R=0.6607, *P*=0.001000. The alpha diversity of gut microbiota in different groups, including **(E)** Ace **(F)** Chao **(G)** Simpson index. (n=5) *p<0.05.

We analyzed the number of common and unique OTUs among the different groups to show that four groups had different compositions (**Figure 5A**). The Shannon index on OTU level indicated that the amount of sequencing data was large enough to reflect the vast majority of gut microbial diversity information in samples (**Figure 5B**). The principal coordinates analysis (PCoA) and non-metric multidimensional scaling (NMDS) with bray_curtis distances on OTU level revealed a distinct clustering of the microbiota composition for each group (**Figures 5C, D**). Furthermore, ACE and Chao index were used to characterize community species richness and Simpson index were used to characterize community diversity. Compared with the OVX, EE could reduce the ACE and Chao index, while improving the Simpson index value. This indicated that EE treatment increased the community diversity of the gut microbiota and diminished community richness (**Figures 5E–G**).

**Figure 6:**
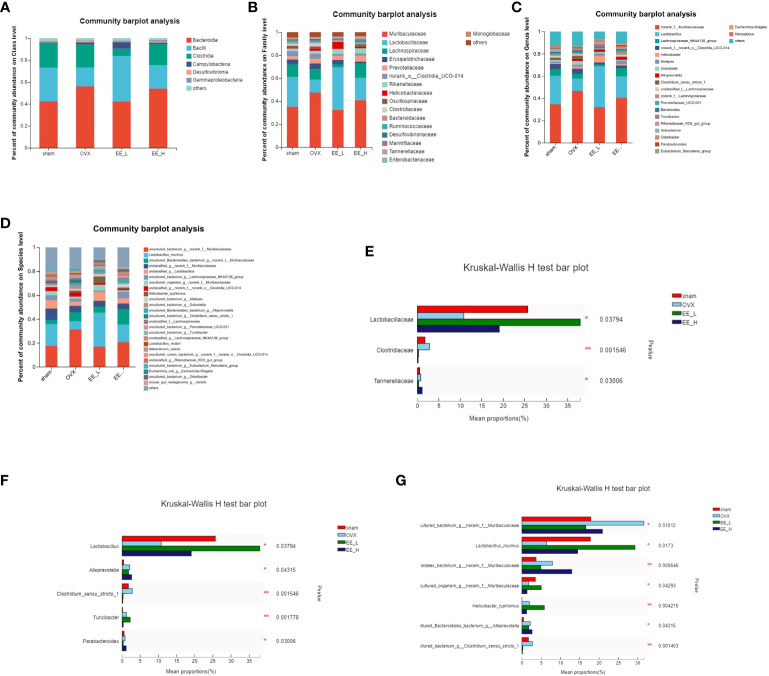
Effects of EE on gut microbiota in OVX mice. Average composition of the **(A)** Class **(B)** Family **(C)** Genus **(D)** Species. Differences in gut microbiota at the **(E)** Family **(F)** Genus **(G)** Species levels. In the column diagram, the red bar represents Sham, the light blue bar represents OVX, the green bar represents EE-L, and the dark blue bar represents EE-H. *p<0.05, **p<0.01.

To study the specific changes in bacterial communities, cluster histograms were drawn to show the changes in gut microbiota at the class, family, genus and species levels in each sample (**Figures 6A–D**). The relative abundances of the dominant bacterial families, genus and species were also compared among the groups (**Figures 6E–G**). At the class level, *Bacteroidia*, *Bacilli*, and *Clostridia* were the predominant in all groups. At the family level, compared to the Sham, OVX reduced the relative abundance of *Lactobacillaceae*, while the relative abundance of *Clostridiaceae* significantly increased. Compared to the OVX, EE increased the relative abundance of *Lactobacillaceae* and decreased the relative abundance of *Clostridiaceae*. At the genus level, OVX decreased the relative abundance of Lactobacillus significantly, while increased the relative abundance of *Clostridium_sensu_stricto_1*. EE treatment increased *Lactobacillus* and decreased *Clostridium_sensu_stricto_1* significantly. At the species level, the contents of *Uncultured_bacterium_g_norank_f_Muribaculaceae* and *Uncultured_bacterium_g_Clostridium_sensu_stricto_1* in the OVX were higher than those in the Sham, whereas the contents of *Lactobacillus_murinus* decreased. Compared to the OVX, EE reduced the relative abundance of *Uncultured_bacterium_g_norank_f_Muribaculaceae* and *Uncultured_bacterium_g_Clostridium_sensu_stricto_1*, while the relative abundance of *Lactobacillus_murinus* significantly increased.

## 4 Discussion

OP is a systemic skeletal disease that results in deteriorated bone structure and low BMD, which can compromise bone strength and increase the risk of fractures (27). Excessive osteoclast formation and enhanced bone loss are the primary contributors to OP incidence (28). Interestingly, the inflammatory response plays a crucial role in regulating OP-related bone remodeling (29). Following menopause, estrogen deficiency increases the expression of pro-inflammatory factors and pro-osteoclastic cytokines, contributing to the development of PMO, the most common type of OP (30). In the present study, we found that the natural compound EE, exerting anti-OP effects via modulating multi-targets and multi-pathways, prevented OVX-induced bone destruction via regulate gut microbiota and inflammatory responses *in vivo*.

Latest advancements in network pharmacology allow researchers to visually analyze disease targets and action pathways, assisting to investigate the mechanism of TCM’s actions (31). The PPI network was constructed by intersecting EE and osteoclast-related targets, generating a network containing 130 functional proteins. The top 10 core targets were TP53, AKT1, JUN, CTNNB1, STAT3, HIF1A, EP300, CREB1, IL1B, and ESR1. TP53, a proapoptotic protein, is closely associated with OP. *In vivo* experiment revealed that mice lacking p53 displayed elevated osteoclastogenesis and bone resorption (32). AKT1 is one of three closely related serine/threonine protein kinases (AKT1, AKT2, and AKT3), which regulates many physiological processes including metabolism, proliferation, cell survival, and angiogenesis (33). A study showed that AKT1 may be a regulator of the differentiation and function of osteoblasts and osteoclasts (34). JUN families include c-Jun is involved in the expression of various inflammatory genes by binding to their transcription factor binding sites. The c-Jun binds to the proximal IL-6 promoter and promotes IL-6 production which has been associated with accelerated osteoclastogenesis and elevated bone resorption (35). CTNNB1 encodes β-catenin which is a key player in the canonical Wnt/β-catenin signaling pathway (36), and has been shown regulate osteoblastic differentiation and osteoclastogenesis (37). In inflammation IL-6 is the major stimulator of STAT3, which plays important roles in inflammation with NF-κB, which expresses IL-6 as a target (38). HIF1A promoted the expression of RANKL by activating JAK2/STAT3 pathway, and enhanced osteocyte-mediated osteoclastic differentiation *in vitro* (39). EP300 is a common transcriptional regulator with a role in both cell proliferation and cell differentiation. EP300 is associated with protein ubiquitination and is up-regulated in trabecular bone samples and osteoblasts from osteoarthritis patients (40). CREB1 and ESR1 are important genes involved in OP pathogenesis (41). IL1B is a proinflammatory mediator. In both mice and humans, IL1B drives Tregs to express RANKL and thereby accelerate osteoclast differentiation (42).

GO and KEGG enrichment analysis are important bioinformatic tools for understanding high-degree relations between genes/proteins and biological processes/signaling pathways (43). GO analysis of the 130 proteins spotted in the previous PPI network showed that EE regulates key cellular processes that control the pathogenesis of OP, such as transcription factor binding, response to hormone, gland development and so on. Intriguingly, KEGG pathway enrichment analysis revealed that EE regulates the Wnt, HIF-1 and Calcium signaling pathways and so forth, hinting that EE acts on multiple targets and pathways during its regulation of OP progression. These findings jointly suggested that EE is a potential therapeutic for OP treatment and the network pharmacology is a reliable tool which can advance our understanding of TCM-mediated mechanisms. Molecular docking studies further provided a visual explanation of the interactions between EE and its predicted protein targets related to OP. Docking analysis provided the first-hand evidence that EE has the good binding activity with the 10 core targets, in which the docking scores of EE with HIF1A have the highest absolute value among the targets.

Previous studies have established connections between intestinal microbiota, gut barrier integrity, chronic inflammation, and the HIF-1 signaling pathway (44). PD’Amelio et al. (45) demonstrated that bone homeostasis is maintained by a healthy composition of microbiota, and gut dysbiosis fuels osteoclast activity and exacerbates OP. This is consistent with the results of network pharmacology and molecular docking. Micro-CT revealed that EE could increasing BMD and restoring the bone trabecular microstructure. In addition, the serum bone resorption indicators TRAP and CTX decreased, while the bone formation indicators P1NP increased after EE intervention. Our results indicated that EE prevents bone destruction in OVX mice due to its inhibitory effect on excessive osteoclastogenesis. *Lactobacillus* supplementation has been shown to benefit human gut microbiota (46, 47), and bone turnover markers (48), providing short-term prevention of bone loss in the lumbar spine. Currently evidence to confirm that the relative abundance of *Clostridiaceae* is negatively correlated with human bone density, and that a decrease in the level of *Clostridiaceae* can prevent bone loss caused by estrogen deficiency (49). Through the observation of gut microbiota in mice, we found that the abundance of *Lactobacillus* was increased and *Clostridiaceae* was decreased by EE treatment, which may be the key mechanism of EE beneficial to OP. A healthy intestinal epithelial barrier can prevent tight hyperpermeability (50), which can filter out abnormally high levels of inflammatory cytokines that lead to hyperactive osteoclasts and excessive bone degradation (51). LPS could damage the intestinal barrier by inducing apoptosis of intestinal cells and reducing inflammatory response, resulting in high intestinal permeability and increased inflammatory response (52). EE reduced the production of LPS and pro-inflammatory cytokines TNF-α and IL-6. Collectively, our results suggested that EE has an impact on gut microbiota and inflammatory response potentially preventing progression of OP.

## 5 Conclusions

In summary, to decipher the molecular mechanisms of EE in the treatment of OP, network pharmacology analysis and molecular docking was performed coupled with *in vivo* validation. The network pharmacology analysis and molecular docking revealed that EE exerted anti-OP effects via modulating multi-targets and multi-pathways, and the *in vivo* results showed that EE regulated gut microbiota and inflammatory responses to alleviate the progression of OP. Of note, our conclusions are only based on limited experimental data, and more comprehensive and in-depth analyses are needed in the future.

## Data availability statement

The datasets presented in this study can be found in online repositories. The name of the repository and accession number can be found below: NCBI; Accession to cite for these SRA data: PRJNA1025301.

## Ethics statement

The Ethics Committee of Guangdong Pharmaceutical University had reviewed and approved the experimental protocol according to the Guide for the Care and Use of Laboratory Animals. The study was conducted in accordance with the local legislation and institutional requirements.

## Author contributions

TZ: Writing – original draft. YZ: Writing – original draft. DG: Writing – review & editing. YX: Writing – review & editing. JW: Writing – review & editing. LT: Writing – review & editing. QD: Writing – review & editing. PS: Writing – review & editing.
